# Survival by race in men with chemotherapy-naive enzalutamide- or abiraterone-treated metastatic castration-resistant prostate cancer

**DOI:** 10.1038/s41391-021-00463-9

**Published:** 2021-11-03

**Authors:** Daniel J. George, Krishnan Ramaswamy, Ahong Huang, David Russell, Jack Mardekian, Neil M. Schultz, Nora Janjan, Stephen J. Freedland

**Affiliations:** 1 Duke Cancer Institute, Duke University School of Medicine, Durham, NC USA; 2 Section of Hematology and Oncology, Durham VA Medical Center, Durham, NC USA; 3 Pfizer Inc., New York, NY USA; 4 Fomerly of STATinMED Research, Plano, TX USA; 5 Astellas Pharma, Inc, Northbrook, IL USA; 6 STATinMED Research, Plano, TX USA; 7 Division of Urology, Department of Surgery, Samuel Oschin Comprehensive Cancer Institute, Cedars-Sinai Medical Center, Los Angeles, CA USA; 8 Section of Urology, Durham VA Medical Center, Durham, NC USA; 9 Present Address: Tigermed, Dallas, TX USA

**Keywords:** Outcomes research, Prostate cancer, Cancer therapy, Prostate cancer, Cancer therapy

## Abstract

**Background:**

Black men are more likely to be diagnosed with aggressive prostate cancer (PC) and die from PC than white men. However, black men with metastatic castration-resistant PC (mCRPC) had longer overall survival (OS) than white men when treated with certain agents in clinical trials. We analyzed claims data from the Veterans Health Administration (VHA) database to evaluate OS in black and white men treated with enzalutamide or abiraterone (novel hormonal therapy [NHT]) for chemotherapy-naïve mCRPC.

**Methods:**

Patients with mCRPC aged ≥18 years were identified in the VHA database by diagnosis codes, evidence of surgical/medical castration, and a prescription claim for enzalutamide or abiraterone after castration from April 2014–March 2017. Cox models assessed associations between race and OS. Unadjusted and multivariable analyses were performed on the entire population and subsets based on the type of therapy received (if any) after NHT.

**Results:**

In total, 2910 patients were identified (787 black, mean 71.7 years; 2123 white, mean 74.0 years). Median follow-up was 19.0 and 18.7 months in blacks and whites, respectively. Black men had better survival versus white men: hazard ratios (95% CIs) were 0.89 (0.790–0.996; *P* = 0.044) and 0.67 (0.592–0.758; *P* < 0.0001) in the unadjusted and multivariable models, respectively. Statistically significantly longer OS was seen in black versus white men regardless of subsequent treatment, including no subsequent treatment.

**Conclusions:**

In the VHA, black men with chemotherapy-naïve mCRPC initiating NHT may have better outcomes than similarly treated white men.

## Introduction

In the United States, population-based statistics for 2020 continue to show that cancer incidence, five-year survival rates, and mortality are worse in African American or black men than in white men [1]. These same trends have been unequivocally evident for prostate cancer (PC) over decades [1–5]. Black men are 1.8 times more likely to be diagnosed with PC and 2.2 times more likely to die of PC than white men [6].

Disparities in PC incidence and mortality by race have been attributed to variations in risk factors, access to care, tumor-related characteristics, and other biologic factors [1, 2, 7]. Black race was associated with 84% greater risk of diagnosis of high-grade disease at the time of biopsy after adjustment for clinical characteristics at the Durham Veterans Affairs Medical Center, Durham, North Carolina, suggesting that factors other than differential access to care are implicated [8]. There is now evidence that differences in tumor cell biology may explain why aggressive and advanced PC is found more frequently in black men at diagnosis [1, 2, 5, 7]. However, detailed real-world analyses that controlled for differences in access to care and known prognostic factors in populations with early-stage PC revealed that the black race was not associated with inferior pathologic, biochemical, or survival outcomes relative to the white race [9, 10]. Among men who received androgen-deprivation therapy for biochemical recurrence after radical prostatectomy, race was likewise not a predictor of metastases or other adverse outcomes [11].

Comparatively less information is available for outcomes among men who progress to advanced PC stratified by race. In clinical trials, black men are regularly under-represented, making analyses inconclusive. Meta-analyses of randomized clinical trials and case-controlled studies suggest that black men with metastatic castration-resistant PC (mCRPC) may have longer overall survival (OS) than white men when treated with chemotherapy or sipuleucel-T [12–14]. In a real-world mCRPC population who received radium-223 dichloride, black men had a lower mortality risk than white men [15]. Thus, while the extant data suggest black men have better OS in the mCRPC setting when treated with some regimens, this has not been well studied for novel hormonal therapies (NHTs) outside of two exploratory studies that found better prostate-specific antigen (PSA) responses in black versus white men treated with abiraterone [16, 17]. Although enzalutamide and abiraterone demonstrated a clear survival benefit in men with mCRPC prior to docetaxel in pivotal clinical trials [18–21], black men constituted <3% of global trial populations [16, 18]. This level of under-representation underlines the need to further analyze survival data for black men with mCRPC in real-world settings.

We therefore hypothesized that among chemotherapy-naïve mCRPC men treated similarly with NHTs, there may be OS differences favoring black men. To test this, we comprehensively assessed OS among black versus white men who received therapy with the two most common first-line treatments for mCRPC–enzalutamide and abiraterone–in equal access, real-world setting.

## Methods

### Study design and oversight

This was a retrospective administrative database analysis conducted to compare OS among black and white men with chemotherapy-naïve mCRPC for which either enzalutamide or abiraterone was prescribed. The study was designed and reported according to best practice guidelines [22] and was exempt from institutional review board approval as it utilized only de-identified claims data.

### Data source

**Fig. 1:**
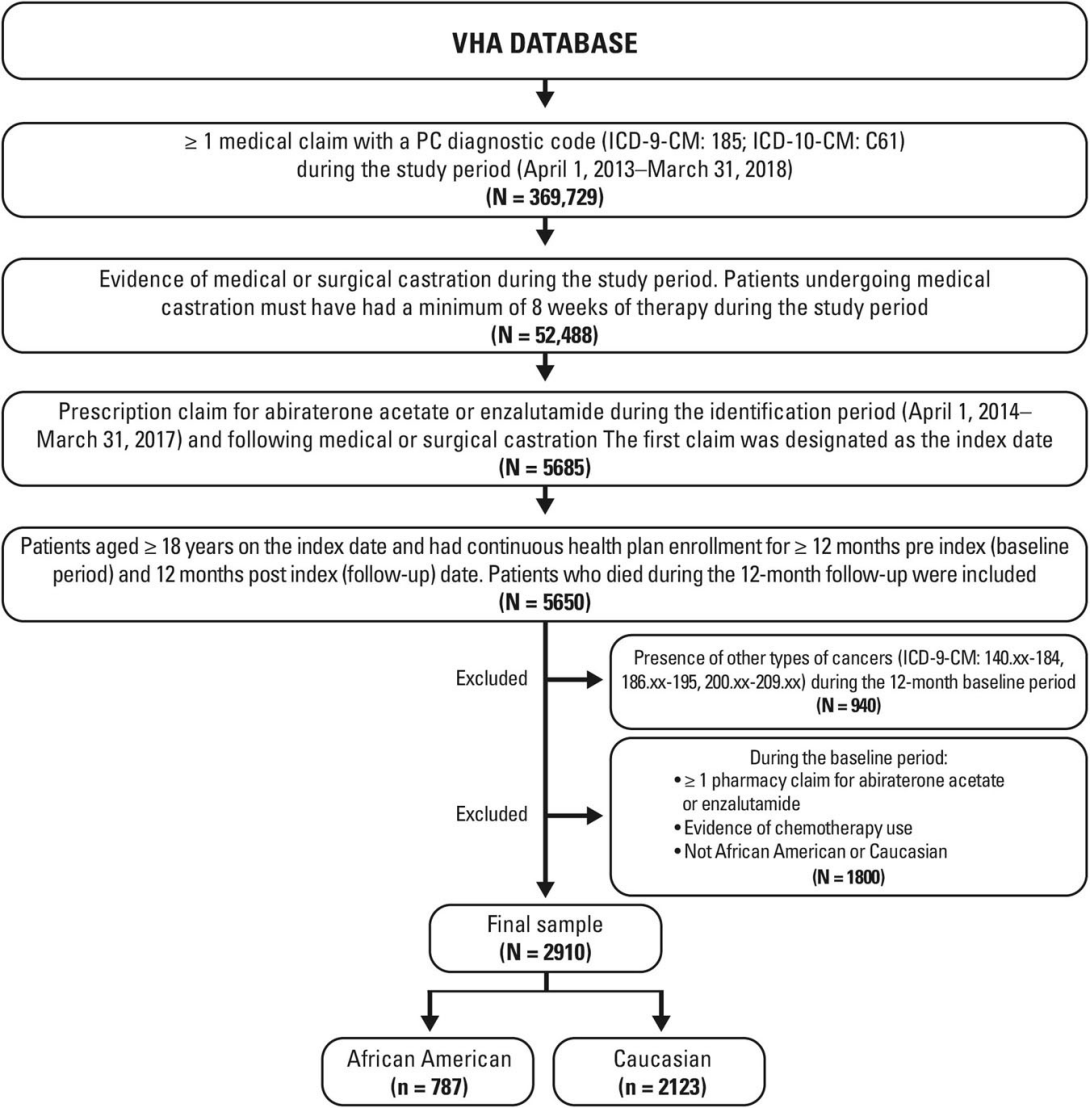
Patient attrition. Abbreviations: *ICD-9-CM/ICD-10-CM* International Classification of Diseases, 9th and 10th Revisions, Clinical Modification, *PC* prostate cancer, *VHA* Veterans Health Administration.

We used data from the US Department of Veterans Health Administration (VHA) database within a five-year study period ranging from April 1, 2013 to March 31, 2018, which comprised a three-year identification period (April 1, 2014–March 31, 2017) and 12-month pre-index (baseline) and post-index (follow-up) periods (Fig. 1). When the cohorts were defined, enzalutamide and abiraterone were indicated only for mCRPC. The VHA is the largest electronically integrated equal access healthcare system in the United States. It serves > 9 million enrolled veterans across the country [23] at 162 VA hospitals and > 850 community and facility-based clinics [24]. The database includes inpatient and outpatient care (provided in appendix) and the vital status file documenting death status and date.

All PC-related diagnoses and procedures were identified by claims with codes from the International Classification of Diseases, 9th and 10th Revisions, Clinical Modification (ICD-9-CM/ICD-10-CM), Current Procedural Terminology, Version 4 procedure codes, and the Health Care Common Procedure Coding System. Mortality is updated quarterly and ascertained from four resources: Social Security Death Master File, Medicare Vital Status Files, VA Beneficiary Identification and Records Locator Subsystem, and the patient treatment file.

### Patient identification

Male patients aged ≥ 18 years with mCRPC were identified based on PC diagnosis code (ICD-9-CM: 185; ICD-10-CM: C61) during the study period, evidence of surgical/medical castration (defined in the appendix; similar to prior studies [25, 26] but with a shorter range for greater inclusivity, ≥ 8 weeks of continued castration therapy was required to define medical castration), and a prescription claim for enzalutamide or abiraterone after castration during the identification period. The index date was defined as the first claim for enzalutamide or abiraterone during the identification period. Race–as reported by the patient, a proxy, or VHA enrollment coordinator or clerk–was extracted from the database to identify the black and white cohorts. Due to limited numbers, patients of other races were not included in this analysis. Hispanic ethnicity was not separately evaluated, but both white and black races studied would have included patients of Hispanic ethnicity.

All patients had continuous VHA enrollment for ≥ 12 months before the index date (baseline period) and after the index date (follow-up period). They were followed until the earliest date of death, disenrollment, or end of study (March 31, 2018). Excluding patients with other cancers (ICD-9-CM: 140.xx-184, 186.xx-195, 200.xx-209.xx) and claims for chemotherapy, enzalutamide, or abiraterone during the 12-month baseline period ensured that study patients were newly initiating mCRPC treatment with enzalutamide or abiraterone.

### Study measures

Patient age (years) was assessed on the index date, and corresponding race information was collected from medical records. Clinical characteristics, including Charlson Comorbidity Index (CCI) score [27, 28], individual comorbidities, and pre-index treatments were measured during the baseline period. Comorbidities of interest included cardiovascular diseases, type 2 diabetes, and liver abnormality/damage (full list provided in appendix).

OS (months) was calculated from the index date to death (regardless of cause) or study end. OS was compared between black and white men over the duration of the study.

Subsequent treatments after index NHT were recorded to capture the extent to which patients received multiple lines of treatment, and to assess the impact on OS.

### Statistical analysis

Descriptive statistics were generated for all study variables–means and standard deviations for normally distributed continuous variables, and medians and interquartile range (IQR) for continuous variables that were skewed. Statistical tests of significance for baseline differences between black and white cohorts were conducted using Χ^2^ tests for categorical variables, *t* tests for normally distributed data, and rank-sum tests for nonparametric data.

The nonparametric Kaplan–Meier product-limit method was used to estimate OS distributions in each cohort. Unadjusted OS was compared between cohorts using hazard ratios (HRs) and 95% CIs, which were estimated using Cox proportional-hazards models. The proportional-hazards assumption was verified using a time-dependent explanatory variable Cox-regression model (*P* = 0.4991). Multivariable analysis was conducted that corrected for differences in patients’ characteristics and the model was built in steps. An initial Cox proportional-hazards model was developed which included the following baseline characteristics: age, baseline comorbidities (including hypertension, acute coronary syndrome/myocardial infarction, stroke, angina pectoris, arrhythmia, congestive heart failure, hyperlipidemia, type 2 diabetes, and liver damage/abnormality), and pre-index long-term corticosteroid use [29]. We concluded that other confounders of mortality could be identified in the VHA dataset and that the model may be strengthened by including these. These included lab values (specifically PSA, alkaline phosphatase, hemoglobin) and claim codes indicating sites of metastases (visceral and bone), which were added to the final Cox model. Separate adjusted OS analyses were conducted for the following subsets: (1) those receiving only first-line treatment with either enzalutamide or abiraterone without any sequential treatment; (2) those who switched from enzalutamide to abiraterone and vice versa; (3) those who switched from first-line enzalutamide or abiraterone to second-line chemotherapy; and (4) those who switched from first-line enzalutamide or abiraterone to other subsequent therapies as well as those receiving > 2 lines of treatment.

All statistical analyses were performed using SAS (v 9.4; SAS Institute Inc., Cary, NC).

## Results

### Patient disposition

A total of 2910 patients met inclusion criteria (Fig. 1), of whom, 787 (27%) were black and 2123 (73%) were white.

### Baseline characteristics

Use of enzalutamide (blacks 40.3%; whites 37.7%) and abiraterone (blacks 59.7%; whites 62.3%) was balanced between groups. At the index date, the mean age of black men with mCRPC was 2 years lower than white men (71.7 and 74.0 years, respectively; Table 1). Black men were more likely to be < 65 years than white men (17.7% vs. 6.6%; *P* < 0.0001), although most men were ≥ 65 years. Mean CCI score was similar between cohorts (6.56 vs. 6.39 in blacks vs. whites, respectively; *P* = 0.23). Overall, a high proportion of both cohorts had at least one comorbid condition and the most prevalent was hypertension. The black cohort had a higher prevalence of hypertension (77.1% vs. 67.1%, *P* < 0.0001), type 2 diabetes (38.1% vs. 29.4%, *P* < 0.0001), and liver damage/abnormality (8.8% vs. 5.2%, *P* = 0.0003) but a lower prevalence of hyperlipidemia (48.3% vs. 54.7%, *P* = 0.0002) relative to the white cohort. PSA level was higher (median 44.6 vs. 26.7 ng/mL; *P* < 0.001) and hemoglobin lower (median 11.8 vs. 12.7 g/dL; *P* < 0.001) in the black versus white cohort. The cohorts were not significantly different with respect to median alkaline phosphatase levels, visceral disease, bone metastasis, or long-term corticosteroid use (8.6% vs. 8.3%, *P* = 0.79).

**Table 1:** Baseline demographic and clinical characteristics of the chemotherapy-naïve metastatic castration-resistant prostate cancer patients by race.

	Black (*n* = 787)	White (*n* = 2123)	*P* Value
Age, mean (SD), y^a^	71.71 (8.41)	74.01 (7.42)	<0.0001
Age category, no. (%)			
18–64	139 (17.66)	139 (6.55)	<0.0001
65–74	286 (36.34)	855 (40.27)	0.0536
75–88	263 (33.42)	809 (38.11)	0.0199
>89	99 (12.58)	320 (15.07)	0.0888
CCI score, mean (SD)	6.56 (3.55)	6.39 (3.50)	0.2303
Comorbidities, no. (%)			
Urinary tract infection	129 (16.39)	189 (8.90)	<0.0001
Impotence	109 (13.85)	131 (6.17)	<0.0001
Cardiovascular			
Hypertension	607 (77.13)	1425 (67.12)	<0.0001
Arrhythmia	68 (8.64)	132 (6.22)	0.0218
Stroke	56 (7.12)	131 (6.17)	0.3557
Congestive heart failure	59 (7.50)	169 (7.96)	0.6793
ACS/MI	19 (2.41)	66 (3.11)	0.323
Angina pectoris	12 (1.52)	46 (2.17)	0.2711
Hyperlipidemia	380 (48.28)	1162 (54.73)	0.0002
Type 2 diabetes	300 (38.12)	624 (29.39)	<0.0001
Liver damage/abnormality	69 (8.77)	110 (5.18)	0.0003
Prognostic variables^b^			
PSA, ng/mL			
Median (IQR)	44.6 (108.4)	26.7 (66.8)	
Mean (SD)	167 (383)	105 (323)	<0.0001
Hemoglobin, g/dL			
Median (IQR)	11.8 (2.4)	12.7 (2.1)	
Mean (SD)	11.6 (1.7)	12.6 (1.8)	<0.0001
ALP, U/L			
Median (IQR)	92 (73)	89 (65)	
Mean (SD)	156 (190)	154 (238)	0.8678
Visceral disease,^c^ no. (%)	23 (2.92)	54 (2.54)	0.5716
Bone metastasis, no. (%)	332 (42.19)	928 (43.71)	0.4605
Pre-index corticosteroids^d,e^	68 (8.6)	177 (8.3)	0.7936

*ACS* acute coronary syndrome, *ALP* alkaline phosphatase, *CCI* Charlson Comorbidity Index, *IQR* interquartile range, *MI* myocardial infarction, *PSA* prostate-specific antigen, *SD* standard deviation.

^a^At the time of the prescription claim for abiraterone acetate or enzalutamide.

^b^Among evaluable patients.

^c^Lung or liver metastasis.

^d^Index treatment was either abiraterone acetate plus prednisone or enzalutamide.

^e^Chronic use for at least three months in the pre-index period.

### Overall survival

The median follow-up time in black patients was 19.0 months (IQR, 17.6; Q1, 12.6; Q3, 30.1) and in white patients was 18.7 months (IQR, 15.0; Q1, 12.5; Q3, 27.5). During this time, 384 black patients (48.8%) and 1105 white patients (52.0%) died.

**Fig. 2:**
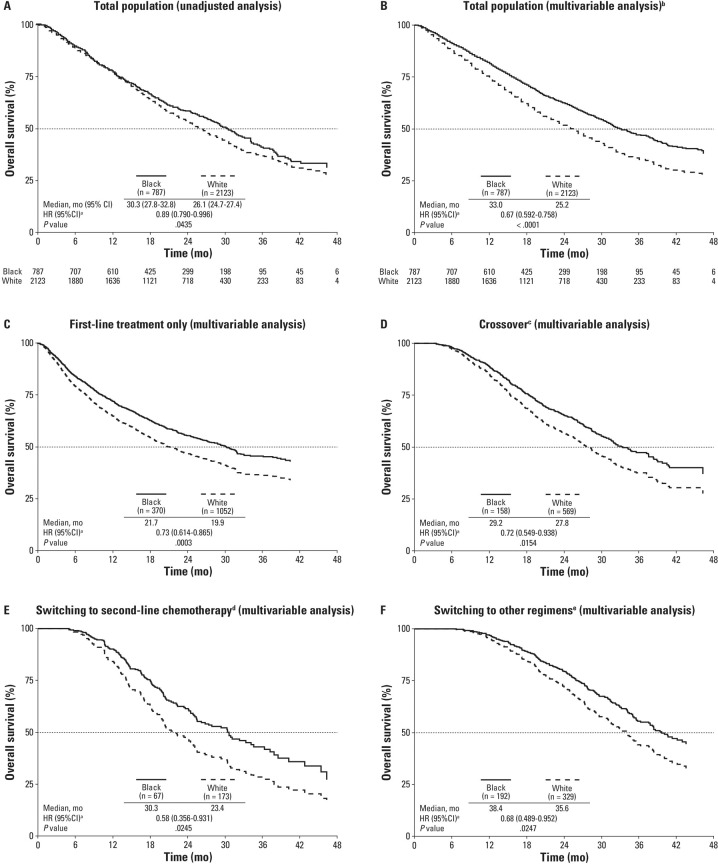
Overall survival. The total population regardless of subsequent treatment (**A**, unadjusted analysis; and **B**, multivariable analysis); individual patient subsets based on subsequent treatment (**C**–**F**). The dashed horizontal lines indicate medians. ^a^Hazard ratio (HR) is based on Cox-regression model with race as the covariable and values of <1.00 favoring black men. ^b^Adjusted for age, comorbidity status, pre-index corticosteroid use, as well as the following established metastatic castration-resistant prostate cancer prognostic variables: prostate-specific antigen, alkaline phosphatase, hemoglobin, and visceral and bone metastasis (ICD-9-CM: 197-199.1). Comorbidity variables in the model included urinary tract infection, impotence, hypertension, acute coronary syndrome/myocardial infarction, stroke, angina pectoris, arrhythmia, congestive heart failure, hyperlipidemia, type 2 diabetes, and liver damage/abnormality. ^c^Switching therapies from enzalutamide to abiraterone and vice versa only. ^d^Chemotherapy-only, including docetaxel, cabazitaxel, or mitoxantrone hydrochloride. ^e^Other = any sequential treatment sequence (including patients who received additional lines of treatment after crossover or chemotherapy as defined above). *ICD-9-CM* International Classification of Diseases, 9th Revision, Clinical Modification.

In the unadjusted analysis, black men had a statistically significantly lower risk of death (11%) versus white men (HR 0.89, 95% CI, 0.790–0.996; *P* = 0.044), with an estimated median OS of 30.3 months (95% CI, 27.8–32.8) among black men and 26.1 months (95% CI, 24.7–27.4) among white men (Fig. 2A). After adjusting for differences in baseline characteristics (age, comorbidities, long-term corticosteroid use, and established mCRPC prognostic variables), risk of death was 33% lower in black men relative to white men (HR 0.67, 95% CI, 0.59–0.76; *P* < 0.0001) with a median OS for black and white men equaling 33.0 and 25.2 months, respectively (Fig. 2B). Table S1 (appendix) provides full results of the multivariable analyses with parameter estimates for the entire population.

Of the 2910 patients in the entire population, 1422 patients (48.9%) received only first-line NHT, 727 patients (25.0%) crossed over from one NHT to the other (i.e., back-to-back NHT), 240 patients (8.2%) switched to second-line chemotherapy, and 521 patients (17.9%) received multiple additional lines of therapy. Fewer black than white patients crossed over from enzalutamide to abiraterone or vice versa (20.1% vs. 26.8%, *P* = 0.0002) while more black than white patients received multiple other lines of therapy (24.4% vs. 15.5%, *P* < 0.0001). The proportion of patients receiving first-line treatment only (47.0% vs. 49.6%, *P* = 0.2237) and receiving second-line chemotherapy only (8.5% vs. 8.2%, *P* = 0.7509) were both similar between black and white patients. Statistically significantly longer OS was detected in black versus white men across all patient subsets stratified by subsequent treatment (Fig. 2C–F).

Overall, the proportion of men who received treatment subsequent to first-line NHT (any line) was similar, with 417 (53.0%) black patients and 1071 (50.4%) white patients receiving additional lines of treatment. Across all lines of treatment, NHTs were the most common subsequent antineoplastic therapy in both black and white men (Table 2).

**Table 2:** Subsequent treatment after initiation of abiraterone and enzalutamide in chemotherapy-naïve metastatic castration-resistant prostate cancer patients by race.^a^

Subsequent treatment (any line)	Black (*n* = 787)	White (*n* = 2123)
	No. of patients (%)	
Received ≥1 antineoplastic treatment	417 (53.0)	1071 (50.4)
Treatments used by ≥5% of patients		
Abiraterone	171 (21.7)	415 (19.5)
Enzalutamide	260 (33.0)	664 (31.3)
Chemotherapy	148 (18.8)	338 (15.9)

^a^The data reported for use of abiraterone, enzalutamide, or chemotherapy in subsequent lines after initiation of abiraterone or enzalutamide are not mutually exclusive. A patient may have had one or more types of the aforementioned therapies in subsequent lines. However, if a patient received the same therapy more than once in subsequent lines, it is counted only once.

Percentages are based on the number of black (*n* = 787) and white (*n* = 2123) patients who received ≥ 1antineoplastic treatment after discontinuation of first-line enzalutamide or abiraterone.

## Discussion

This retrospective cohort study was performed to assess the prognostic significance of race among men treated with NHTs as first-line androgen-targeted therapy for chemotherapy-naïve mCRPC in equal access, real-world setting. Our results show that black men had a significantly lower risk of death than white men on unadjusted (11% risk reduction) and multivariable (33% risk reduction) analyses that accounted for age, comorbidities, and mCRPC prognostic factors. Consistent survival differences between black and white men (27–42% risk reduction) were seen regardless of subsequent treatments, including no subsequent treatment.

The longer OS detected among black men with chemotherapy-naϊve mCRPC treated with NHTs relative to white men aligns with improved outcomes in black versus white men in clinical trials of other life-prolonging agents [12, 13, 16, 17] and a small real-world study [15]. However, our analysis is the first and largest to date to evaluate real-world OS outcomes by race in chemotherapy-naϊve mCRPC men treated with NHTs. In the larger of two pooled analyses of data from randomized phase 3 trials involving 8820 men with mCRPC treated with chemotherapy [12, 30], black men had a 19% reduction in risk of all-cause death relative to white men (multivariable HR, 0.81; 95% CI, 0.72–0.91; *P* < 0.001) [12]. Similarly, black men had longer survival than white men when treated with sipuleucel-T in an exploratory analysis of three phase 3 trials of advanced PC (HR, 0.735; 95% CI, 0.613–0.882; *P* < 0.001) [13], which was confirmed in the PROCEED registry of men with mCRPC (HR, 0.81, 95% CI, 0.68–0.97; *P* = 0.03) [14]. In a small, heterogeneous VHA cohort of 318 men with mCRPC who received radium-223 dichloride, black men had a 25% lower mortality risk than white men on multivariable analysis (HR, 0.75; 95% CI, 0.57–0.99; *P* = 0.045) [15]. A prospective clinical trial showed longer trends in time to PSA progression and greater PSA response rates with abiraterone in black versus white men, though likely due to limited power no differences in survival were seen [17]. Nevertheless, it shows the importance of conducting prospective studies in different races to confirm real-world evidence seen regarding differential outcomes across races.

Multiple independent data sets from clinical trials and real-world populations of mCRPC patients involving different therapeutic interventions with different mechanisms of action, have demonstrated improved survival for black men. Whether this reflects improved treatment responses requires further prospective study, as does the elucidation of any mechanism(s) underpinning potential racial differences in response to different mCRPC therapies. Specifically, whether differences in efficacy are driven by ancestry-associated genetic or other biologic or nonbiologic determinants. Such understanding could help select patients for NHT earlier and maximize disease control.

The median OS of the black cohort in this large, chemotherapy-naïve mCRPC population (black men, 30 months and white men, 26 months, with over 19 months of follow-up) is consistent with OS among the overall population of patients who received enzalutamide (PREVAIL; 35.3 months over 31 months of follow-up) and abiraterone (COU-AA-302; 34.7 months over 49.2 months of follow-up) in the pre-chemotherapy mCRPC setting [19, 21]. These results suggest that the care of patients in this large single-payer health network is comparable to medical centers globally that enrolled patients in these phase 3 trials, recognizing that clinical trial patients tend to have fewer comorbidities than patients in the real-world setting because of eligibility criteria restrictions [31]. Such results support broadening inclusion criteria for phase 3 trials to allow patients with comorbidities greater access. The patient population was also consistent with other mCRPC studies, as black men were younger and had more unfavorable prognostic factors than white men [12, 15, 32, 33]. These baseline differences are unlikely due to differential screening practices, as the VHA is an equal access system.

Our findings have noteworthy implications for PC management. First, while there is evidence from randomized controlled clinical trials that NHTs have benefits in both black and white men, black men are under-represented in the pivotal trials that led to regulatory approvals [18, 20]. While the design of the current study (in the absence of a control arm) prohibits direct conclusions about the benefit of NHTs in black men, the median OS (for both groups) is roughly consistent with the overall OS benefit observed in phase 3 trials [19, 21]. While the greater OS in this study among black versus white men could be explained by an imbalance of prognostic factors not assessed, we have no evidence to support this from the prognostic factors that were available. An alternative, speculative explanation requiring further validation could be that black men derive a greater benefit from NHTs in the pre-chemotherapy setting. Of note, while we saw NHT to NHT crossover in more white than black patients and we saw other lines of therapy in more black than white patients, the directionality of the OS difference was not affected. In addition, there were no significant racial differences in the overall proportion receiving abiraterone or enzalutamide in any line, and only small differences in the overall proportion of patients who received at least one subsequent treatment and chemotherapy (any line) following index NHT, suggesting OS differences in the overall population was not driven by subsequent treatment.

Comprehensive clinical information is not available in administrative claims; thus, the study identified patients with mCRPC based on prescription claims for enzalutamide or abiraterone after castration, because during the index period (April 2014–March 2017) both drugs were indicated only for mCRPC. We therefore cannot exclude the possibility that some men were hormone-sensitive, though the number is likely low. Regardless, there are no reasons to believe that black or white men would be more likely to receive these agents for hormone-sensitive disease. Furthermore, due to the time-lag in the availability of VHA data for this study (April 1, 2013–March 31, 2018), results may not completely reflect current practice as more treatments and some subsequent lines of therapy are now available for mCRPC. However, although this treatment availability may prolong survival in both black and white men, there should be no bias in relative treatment effect as the VHA is an equal access system. Further analysis in the future can be considered to assess more current outcomes within this population.

Results of this analysis may not be fully generalizable to other US populations with mCRPC, including those with commercial insurance. Our study focused only on black and white races as self-reported in the VHA dataset and did not include men of other races and ethnicities. Further research is recommended to understand outcomes in these groups. Although most patients made prescription claims over several months, a claim does not constitute therapy administration. In addition, certain tumor burden covariables such as Gleason score and number of metastases, which could have influenced OS, were not available in our dataset.

## Conclusions

In this analysis of real-world claims data, black men had improved OS relative to white men treated with enzalutamide or abiraterone for chemotherapy-naïve mCRPC in a single-payer system with equal access, and adjusting for relevant covariables. Further research is needed to understand whether the response to therapy by race may be due to underlying molecular/genomic drivers.

## Supplementary information

APPENDIX Survival by race in men with chemotherapy-naïve enzalutamide- or abiraterone-treated metastatic castration-resistant prostate cancer
