# Supplementary material for: Survival by race in men with chemotherapy-naive enzalutamide- or abiraterone-treated metastatic castration-resistant prostate cancer
Source: Prostate Cancer Prostatic Dis. 2021 Nov 3;25(3):524–30. doi: 10.1038/s41391-021-00463-9 (PMC9385484; doi:10.1038/s41391-021-00463-9)
Supplement: Supplementary file 1 — APPENDIX Survival by race in men with chemotherapy-naïve enzalutamide- or abiraterone-treated metastatic castration-resistant prostate cancer [file 41391_2021_463_MOESM1_ESM.docx]

# APPENDIX

Survival by race in men with chemotherapy-naïve enzalutamide- or abiraterone-treated metastatic castration-resistant prostate cancer

Daniel J. George, MD, Krishnan Ramaswamy, PhD, Ahong Huang, MS, David Russell, MD, Jack Mardekian, PhD, Neil M. Schultz, PharmD, Nora Janjan, MD, MPSA, MBA, Stephen J. Freedland, MD

## Data source

The Veterans Health Administration (VHA) database includes information on four inpatient datasets (main, bed section, procedure, and surgery), one outpatient file (events), the Decision Support System (DSS) costs, and two DSS Clinical National Data Extract files (laboratory results and pharmacy).

## Patient identification

Surgical castration was defined based on Current Procedural Terminology (54520, 54522, 54530, 54535, 54690), International Classification of Diseases, 9th Revision, Clinical Modification (ICD-9-CM) procedure codes (62.3, 62.41, 62.42), and ICD-9-CM diagnosis codes (V45.77). Medical castration was defined based on Health Care Common Procedure Coding System codes (J1675, J1950, J3315, J9202, J9217, J9218, J9219, J9225, J9226, S0175, S9560) and national drug codes for luteinizing hormone-releasing hormone agonists (leuprolide, goserelin, triptorelin, and histrelin implant) or antiandrogens (bicalutamide, flutamide, nilutamide).

Table S1 Multivariable Cox model for overall survival among chemotherapy-naïve, enzalutamide- and abiraterone-treated patients with metastatic castration-resistant prostate cancer adjusted for age, comorbidity status, and pre-index corticosteroid use, as well as prognostic variables

| **Covariable** | **Parameter estimate** | **Standard error** | **Chi-square** | **Pr > Chi-square** | **Hazard Ratio**  **(95% CI)** |
| --- | --- | --- | --- | --- | --- |
| **Age, years** |  |  |  |  |  |
| 18-64 | Reference | | | | |
| 65-74 | 0.03 | 0.10 | 0.07 | 0.7979 | 1.03 (0.84-1.25) |
| 75-88 | 0.25 | 0.10 | 5.89 | 0.0152 | 1.28 (1.05-1.56) |
| >89 | 0.49 | 0.11 | 19.82 | <.0001 | 1.64 (1.32-2.04) |
| **Individual comorbidities** |  |  |  |  |  |
| Urinary tract infection | 0.56 | 0.08 | 56.22 | <.0001 | 1.76 (1.52-2.04) |
| Impotence | -0.14 | 0.11 | 1.76 | 0.1851 | 0.87 (0.71-1.07) |
| Hypertension | 0.10 | 0.06 | 2.81 | 0.0938 | 1.11 (0.98-1.25) |
| Arrhythmia | -0.001 | 0.10 | 0.00 | 0.992 | 1.00 (0.82-1.21) |
| Stroke | 0.35 | 0.10 | 13.43 | 0.0002 | 1.42 (1.18-1.72) |
| Congestive heart failure | 0.26 | 0.09 | 8.91 | 0.0028 | 1.30 (1.10-1.55) |
| ACS/MI | 0.22 | 0.14 | 2.30 | 0.1297 | 1.24 (0.94-1.64) |
| Angina pectoris | -0.22 | 0.20 | 1.25 | 0.2626 | 0.80 (0.54-1.18) |
| Hyperlipidemia | -0.07 | 0.06 | 1.82 | 0.1773 | 0.93 (0.83-1.03) |
| Type 2 diabetes | -0.07 | 0.06 | 1.22 | 0.2689 | 0.94 (0.84-1.05) |
| Liver damage/abnormality | -0.10 | 0.11 | 0.76 | 0.3825 | 0.91 (0.73-1.13) |
| Pre-index corticosteroids^a^ | 0.14 | 0.09 | 2.43 | 0.1192 | 1.15 (0.96-1.38) |
| **Prognostic variables** |  |  |  |  |  |
| PSA |  |  |  |  |  |
| Q1 (≤10.99 ng/mL) | Reference | | | | |
| Q2 (10.99-30.5 ng/mL) | 0.16 | 0.09 | 3.60 | 0.0578 | 1.18 (1.00-1.40) |
| Q3 (30.5-90 ng/mL) | 0.34 | 0.08 | 16.42 | <.0001 | 1.41 (1.19-1.66) |
| Q4 (>90 ng/mL) | 0.69 | 0.08 | 68.28 | <.0001 | 2.00 (1.70-2.36) |
| Missing | 0.23 | 0.18 | 1.71 | 0.1908 | 1.26 (0.89-1.78) |
| Hemoglobin |  |  |  |  |  |
| Q1 (≤11.3 g/dL) | Reference | | | | |
| Q2 (11.3-12.5 g/dL) | -0.30 | 0.07 | 18.18 | <.0001 | 0.74 (0.65-0.85) |
| Q3 (12.5-13.5 g/dL) | -0.59 | 0.08 | 55.69 | <.0001 | 0.55 (0.47-0.65) |
| Q4 (>13.5 g/dL) | -0.66 | 0.08 | 61.91 | <.0001 | 0.52 (0.44-0.61) |
| Missing | -0.55 | 0.13 | 17.51 | <.0001 | 0.58 (0.45-0.75) |
| Alkaline phosphatase |  |  |  |  |  |
| Q1 (≤68 IU/L) | Reference | | | | |
| Q2 (68-90 IU/L) | 0.22 | 0.08 | 6.56 | 0.0104 | 1.24 (1.05-1.47) |
| Q3 (90-136 IU/L) | 0.38 | 0.08 | 20.97 | <.0001 | 1.47 (1.25-1.73) |
| Q4 (>136 IU/L) | 0.90 | 0.08 | 119.35 | <.0001 | 2.46 (2.09-2.89) |
| Missing | 0.69 | 0.16 | 17.73 | <.0001 | 2.00 (1.45-2.76) |
| Visceral disease^b^ | 0.49 | 0.14 | 11.85 | 0.0006 | 1.64 (1.24-2.17) |
| Bone metastasis | 0.06 | 0.05 | 1.39 | 0.239 | 1.07 (0.96-1.19) |
| **Race** |  |  |  |  |  |
| White | Reference | | | | |
| Black | -0.40 | 0.06 | 39.93 | <.0001 | **0.67 (0.59-0.76)** |

^a^Chronic use for at least 3 months in the pre-index period.

^b^Lung or liver metastasis.

*ACS*: acute coronary syndrome, *MI*: myocardial infarction.
